# Surgical Risk and Revascularization—An Update

**DOI:** 10.1016/j.jscai.2026.104392

**Published:** 2026-03-12

**Authors:** Guillaume Marquis-Gravel, Guangyu Tong, Matthew Dodd, Tim Clayton, Matthew Ryan, Kieran F. Docherty, Alicia Williams, Jiaxuan Sun, Stephen E. Fremes, Alexandra J. Lansky, Eric J. Velazquez, Divaka Perera, Mark C. Petrie, Jean-Lucien Rouleau

**Affiliations:** Department of Medicine, Montreal Heart Institute, Université de Montréal, Montreal, Canada; Yale School of Medicine, New Haven, Connecticut; Department of Medical Statistics, London School of Hygiene and Tropical Medicine, London, United Kingdom; Department of Medical Statistics, London School of Hygiene and Tropical Medicine, London, United Kingdom; Kings College London, British Heart Foundation Centre of Research Excellence, St Thomas’ Hospital, and Cardiovascular Division, Guy’s and St Thomas’ NHS Foundation Trust, London, United Kingdom; School of Cardiovascular and Metabollic Health, University of Glasgow, Glasgow, Scotland, United Kingdom; Yale School of Public Health, New Haven, Connecticut; Yale School of Public Health, New Haven, Connecticut; Department of Surgery, Sunnybrook Health Sciences Centre, University of Toronto, Toronto, Canada; Yale School of Medicine, New Haven, Connecticut; Yale School of Medicine, New Haven, Connecticut; Kings College London, British Heart Foundation Centre of Research Excellence, St Thomas’ Hospital, and Cardiovascular Division, Guy’s and St Thomas’ NHS Foundation Trust, London, United Kingdom; School of Cardiovascular and Metabollic Health, University of Glasgow, Glasgow, Scotland, United Kingdom; Department of Medicine, Montreal Heart Institute, Université de Montréal, Montreal, Canada

Since the publication of our manuscript, “Surgical risk and long-term mortality with percutaneous coronary intervention (PCI) and coronary artery bypass grafting (CABG) in ischemic left ventricular systolic dysfunction,” in the September 2025 issue of the *Journal of the Society for Cardiovascular Angiography & Interventions*,^1^ a number of important points have been brought up about the appropriateness of using surgical risk score to stratify prognosis after PCI, the impact of recent improvements in optimal medical therapy (OMT) on the treatment effect of revascularization, and the repercussions of completeness of revascularization in our article. In this letter, we would like to address these issues and briefly discuss areas of continued research.

The extent of coronary disease complexity, which can be assessed with tools designed specifically for patients undergoing PCI such as the SYNTAX score II, represents a meaningful predictor of clinical outcomes. SYNTAX score II, however, was derived from a population that included an extremely small number of patients with heart failure or low left ventricular (LV) ejection fraction. On the contrary, surgical risk scores such as EuroSCORE II serve as complementary tools (when used in addition to a thorough clinical evaluation) to stratify surgical risk and support decision making regarding the most appropriate revascularization modality. Indeed, clinical practice guidelines provide different recommendations regarding PCI or CABG in patients with and without high baseline surgical risk. The EuroSCORE II has previously been shown to effectively evaluate operative risk in the STICH trial.^2^ By showing that this score and important clinical variables differed significantly between the STICH and REVIVED-BCIS 2 trials, our findings illustrate that the 2 trials represent 2 distinct clinical phenotypes that cannot be compared directly. This represents a key finding that enables a more informed interpretation of the clinical implications of both trials. Although not specifically evaluated, it is probable that a large proportion of patients in REVIVED-BCIS 2 were not deemed amenable to CABG before being approached for randomization to PCI vs OMT alone, further differentiating this population from that of the STICH trial. Using EuroSCORE II in our analysis was useful to identify a high surgical risk population that could have potentially benefited from PCI, which was not the case in REVIVED-BCIS 2. By demonstrating this baseline difference in surgical risk between both trials, our study showed that the question regarding the role of PCI in low-to-moderate surgical risk patients with multivessel disease and LV dysfunction remains wide open. The next step will be to evaluate the treatment of choice for patients deemed operable and amenable to PCI, which is currently being studied in multicenter randomized trials.^3^^,^^4^

The influence of contemporary OMT is crucial in determining prognosis and needs to be accounted for in comparing 2 trials conducted more than 10 years apart. Our group recently conducted another combined analysis of the STICH and REVIVED-BCIS 2 trials specifically addressing this question.^5^ In brief, it showed that patients randomized to the OMT group of the STICH trial had a significantly higher risk of all-cause mortality or heart failure hospitalization compared with those randomized to the OMT group in the REVIVED-BCIS 2 trial, lending credence to the hypothesis that improvements in medical therapy between the 2 eras had a strong prognostic impact. Patients randomized to CABG in STICH also experienced more primary events than those randomized in either group in the REVIVED-BCIS 2 trial. In our study, we decided to conduct the analyses separately within each study to allow comparability of the treatment group with its own control group, including regarding baseline pharmacotherapy. This approach minimizes crosstrial confounding, thereby ensuring that the conclusion regarding the lack of interaction between baseline surgical risk and the treatment effect of PCI or CABG remain valid within each study population. Sodium-glucose transport 2 inhibitors were not routinely used at the time the trials were conducted, and rates of clinical outcomes with OMT would likely be lower in a more contemporary cohort, potentially reducing the treatment effect of revascularization.

Finally, completeness of revascularization impacts prognosis. In the trials from the STICH 3.0 International Trial Consortium,^4^ in which patients with LV dysfunction and multivessel disease are randomized to CABG or PCI, complete revascularization needs to be attempted in all participants. In addition, best practices in PCI and CABG are recommended to ensure state-of-the art contemporary revascularization techniques are used, enhancing generalizability of the results.

## Peer review statement

Editor-in-Chief Alexandra J. Lansky had no involvement in the peer review of this article and has no access to information regarding its peer review. Full responsibility for the editorial process for this article was delegated to Deputy Editor Dean J. Kereiakes.
